# Theta Oscillations and Reactivity of Hippocampal Stratum Oriens Neurons

**DOI:** 10.1100/tsw.2010.90

**Published:** 2010-05-18

**Authors:** Valentina F. Kitchigina

**Affiliations:** Institute of Theoretical and Experimental Biophysics and Pushchino State University, Puschino, Moscow region, Russia

**Keywords:** theta rhythm, putative hippocampal interneurons, selection of signals, responses to stimulation, reset, phase locking to stimulus

## Abstract

The supposition was advanced that the neuronal theta rhythmicity is the key mode of signal selection at the hippocampal level. To address this hypothesis, the experimental data on the responses of putative hippocampal interneurons of the stratum oriens CA1-CA3 to stimulation during enhanced theta rhythm and after its blockade are reviewed. Both a strong increase and a decrease of the natural theta rhythm disturbed the reactions of hippocampal neurons; during theta augmentation, the responses were masked or disappeared, and after theta blockade, they lost the ability to habituate. In both cases, two important events were broken: the resetting of the background activity and the phase-locking of theta cycles to stimulus. These data allow one to suppose that only important stimuli are normally capable to evoke these events and these stimuli are selected for recording. When the response to a significant stimulus occurs, the following theta prevents the responses to other stimuli. This probably protects the hippocampal activity from interference from irrelevant signals. Presumably, the absence of the theta deprives the hippocampus of this protection. During enhanced and persistent theta oscillations, the reset disappeared and theta bursts were generated without stimulus locking. In this state, the system is probably closed and the information cannot be recorded. During the theta blockade, the reset was too long and did not habituate. In this case, the system is open for any signals and the hippocampus loses the ability to select signal. This analysis suggests that information selection in the hippocampus may be performed with the participation of nonpyramidal neurons.

## INTRODUCTION

Theta oscillations are the major operational mode of the hippocampus[3]; therefore, they are often regarded as a key to the interpretation of hippocampal functions[3,5,15,29,33,38]. In the last decade, close links have been established between theta oscillations and a variety of cognitive functions. These specific links include the correlation of theta band oscillations with the phase encoding of spatial information in the hippocampus[32] and the formation of mnemonic neuronal representation[19]. Numerous

*The author dedicates this paper to the memory of her mentor, Olga S. Vinogradova, in connection with the 80th anniversary of her birth.
©2010 with author.
Published by TheScientificWorld; www.thescientificworld.com investigations have shown that theta rhythm is relevant to processes of human learning and memory[2,6,16,19,26,34,38]. Undoubtedly, these cognitive functions demand some mechanisms for switching the attention associated with the selection of input information. Recently, it has been proposed that various neuronal oscillations serve as crucial instruments of active input selection at the level of the primary sensory cortex[37]. The hypothesis was advanced that the neuronal theta rhythmicity determines the signal selection at the level of the hippocampus. The experimental data on the responses of hippocampal cells to sensory and electrical stimulation under control conditions and during artificially increased or blocked theta rhythm are reviewed here with emphasis on the responses of putative interneurons of the CA1-CA3 hippocampal stratum oriens[22,23,24,43,44,45,46,47]. A subset of these cells directly contributes to the theta rhythm generation; they are tuned to operate at theta frequencies[13,14,25], and the disruption of their spiking is accompanied by a loss of both the field theta and IPSP trains at the theta frequency[12].

## THE CRITERIA FOR DATA SELECTION

Many types of inhibitory cells have been detected in stratum oriens; among “horizontal” interneurons only, eight varieties of units are distinguished[9,31]. In this analytic review, putative hippocampal interneurons have been identified on the basis of their coordinates and the firing pattern[4,7,27]. In particular, these cells typically fired at high rates (>5 imp/sec) and discharged in rhythmic groups at the field theta frequency. These cells will be called hereafter theta-interneurons or theta cells because their activity exhibited theta modulation. In these theta cells, the baseline activity was analyzed in order to determine the average spiking rate and rhythmicity. The analysis involved the computation of autocorrelation and spectral histograms, and the time constant of theta extinction (“rhythmicity index”, $\tau_\theta$), which characterize the degree of theta stability[23].

The data were obtained on chronic unanesthetized, noncurarized, waking rabbits slightly restricted in their movements. The sensory and electrical stimulations were applied in the control and after the artificial adjustments of theta rhythm. An increase in the hippocampal theta rhythm was achieved by three different methods: (1) an intravenous injection of the acetylcholinesterase inhibitor physostigmine[43,46], (2) an intraventricular injection of the alpha2 adrenoceptor agonist clonidine[23,24], or (3) an injection of the local anesthetic lidocaine into the median raphe nucleus[22,41] (see section “Strong Increase of Theta Rhythm Blocks the Majority of Responses”). In the same or separate experiments, the theta was blocked by three methods: (1) an intravenous injection of the antagonist of cholinergic muscarinic receptors scopolamine[43,46], (2) an injection of lidocaine into the medial septal-diagonal band region (MS)[47], or (3) an intraventricular injection of the alpha2 adrenoceptor antagonist idazoxan[23] (see section “Theta Rhythm Blockade Preserves and Enhances the Responses to the Input Stimulation”).

Each method had some specific effects that will not be discussed here. Instead, the paper will focus on the most common features of theta interneurons’ baseline activity and responses to stimuli under all three increased theta conditions, and all three decreased theta conditions, to compare these to the controls in the same animals.

## CHARACTERISTICS OF THE BASAL ACTIVITY AND EVOKED RESPONSES OF STRATUM ORIENS THETA INTERNEURONS UNDER CONTROL CONDITIONS

The data presented in the review are based on the analysis of the activity of 185 putative theta interneurons of CA1-CA3 stratum oriens that exhibited theta modulation. These theta cells were selected from a pool of hippocampal neurons recorded in 20 rabbits[22,23,24,43,44,45,46,47].

## Basal Activity

During quiet waking, stratum oriens theta cells showed different degrees of theta modulation; from a weak episodic expression to continuous rhythmic bursts (Fig. 1A). The largest number of cells (54.4%) exhibited unstable theta modulation with the rhythmicity index ($\tau_\theta$) ranging from 0.09 to 0.30 sec. The rest of the theta interneurons (45.6%) showed a relatively regular theta activity with $\tau_\theta$ from 0.31 to 0.50 sec (mean value: 0.29 ± 0.06 sec). The bursting frequency varied from 4.5 to 6.05 Hz (mean: 5.25 ± 0.18 Hz). The duration of average theta bursts was 85.6 ± 7.1 msec with 3.9 ± 0.5 spikes in the burst (see all the parameters in Table 1). The simultaneously recorded hippocampal field activity represented mainly theta, delta, and beta waves (Fig. 1A,C). The frequency of field theta oscillations corresponded to average frequency of theta bursts and ranged from 4.25 to 6.15 Hz (mean: 5.20 ± 0.16 Hz) (Table 1).

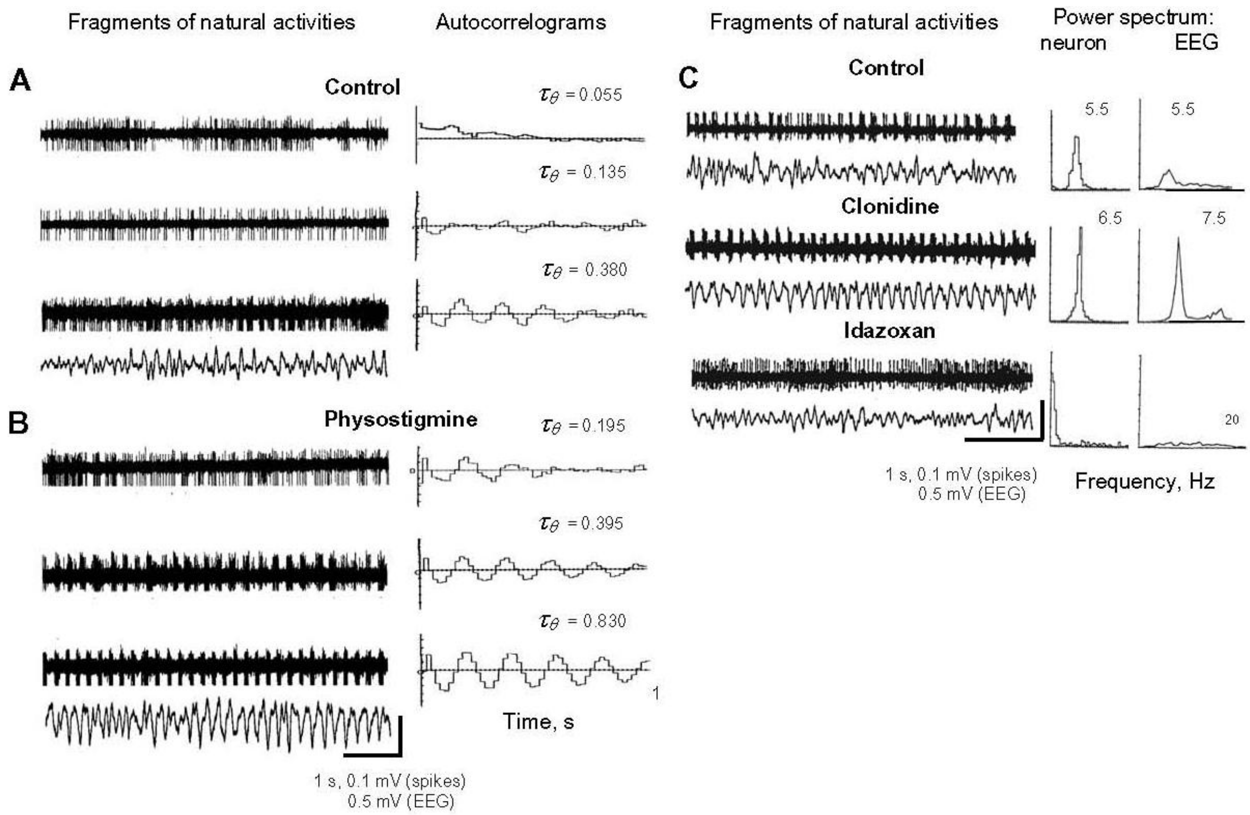

**FIGURE 1.** Theta activity of hippocampal CA1-CA3 stratum oriens putative interneurons in control and during artificially changed hippocampal theta rhythm. Theta bursting interneurons in control (A) and in enhanced theta state caused by physostigmine (B). To the left: neuronal pattern of discharges with different values of rhythmicity index ($\tau_\theta$) (above) and hippocampal EEG (below). To the right: autocorrelation histograms (X axis – time, sec; Y axis – the number of spikes/bin); a number above each histogram indicates the rhythmicity index. (C) The activity of the same theta interneuron (left) in control, high theta (alpha2 adrenoceptor agonist clonidine) and low theta conditions (alpha2 adrenoceptor antagonist idazoxan). To the right: spectral histograms for the neuron and EEG (X axis – frequency, Hz, bin 0.5 Hz; Y axis – power of the rhythmic process, relative units).

## Responses to Sensory Stimulation

Sensory stimuli of various modalities were applied in a series of eight to 15 with intervals 10 sec between them. Auditory (pure tones, whistle, click), visual (light flash, general illumination of experimental chamber), and somatosensory (air puff to vibrissae) stimuli were used. From two to four different series

**TABLE 1**
**Characteristics of Theta Activity of Hippocampal Neurons in the Control and After Injections of Drugs (Mean ± S.E.M.)**

| Characteristics | Control | Physostigmine | Clonidine | Lid into MR | Scopolamine | Idasoxan | Lid into MS |
|---|---|---|---|---|---|---|---|
| Neurons: | | | | | | | |
| Overall firing rate (spikes/sec) | 17.8 ± 1.4 | 18.2 ± 2.5 | 32.3 ± 2.8** | 21.3 ± 1.8 | 16.4 ± 1.2 | 12.2 ± 1.2* | 12.9 ± 2.2 |
| Frequency of bursts (Hz) | 5.25 ± 0.18 | 5.2 ± 0.1 | 6.8 ± 0.25** | 5.5 ± 0.2 | 5.3 ± 0.1 | 4.75 ± 0.11** | — |
| Regularity of bursts, $\tau_\theta$ (sec) | 0.29 ± 0.06 | 0.45 ± 0.04** | 0.61 ± 0.06** | 0.42 ± 0.02** | 0.19 ± 0.02** | 0.20 ± 0.03* | — |
| Duration of bursts (msec) | 85.6 ± 7.1 | 81.4 ± 3.0 | 79.9 ± 3.9* | 81.8 ± 5.1 | 82.4 ± 5.9 | 94.1 ± 8.1 | — |
| Duration of pauses (msec) | 91.5 ± 7.2 | 96 ± 4.6 | 68.9 ± 4.2* | 93.2 ± 3.9 | 94.7 ± 8.1 | 99.7 ± 8.1 | — |
| Number of spikes per burst | 3.9 ± 0.5 | 4.3 ± 0.2 | 5.9 ± 0.4** | 5.4 ± 0.3* | 3.8 ± 0.4 | 3.5 ± 0.35 | — |
| EEGs: | | | | | | | |
| Theta band amplitude (%) | 100 | 195 ± 82** | 490 ± 89** | 180 ± 89** | 72 ± 12* | 51 ± 12** | — |
| Frequency of theta (Hz) | 5.20 ± 0.16 | 5.25 ± 0.1 | 6.8 ± 0.25** | 5.4 ± 0.15 | 5.25 ± 0.15 | 4.9 ± 0.12 | — |
| Number of tests | 185 | 26 | 15 | 28 | 26 | 16 | 28 |

Abbreviations: Lid, lidocaine; MR, median raphe nucleus; MS, medial septal-diagonal band region.

Statistical analysis was performed using one-way ANOVA followed by post-hoc test with appropriate Bonferroni adjustments in the indicated number of tests (n): significant effects present at *$p < 0.05$ or **$p < 0.01$ vs. control parameters.

of stimuli were tested in a single neuron in the control state. The stimuli evoked different responses in different theta interneurons; inhibitory and excitatory reactions dominated (51 and 39%, respectively). As a rule, the responses to different stimuli were alike in the same theta cell, but in a few theta interneurons (in the CA1 field), stimuli-specific responses were observed. Among the excitatory responses, patterned on-responses were sometimes observed, which consisted of one or two dense theta bursts. Usually, the inhibitory and excitatory reactions were accompanied by an increase in regularity of theta (Fig. 2A,B). The frequency of discharges in some cells (10%) did not change, but they demonstrated a distinct increase in theta modulation (Fig. 2C). A most common feature of sensory responses was the initial short (108.6 ± 4.7 msec) inhibition of the theta activity, although it could be more clearly seen in excitatory responses (Fig. 2B); inhibitory responses tended to overlap this short inhibition (Fig. 2A). In the majority of cells (82%), inhibitory and excitatory neuronal responses to sensory stimuli decreased or disappeared (habituated) after 10 to 12 repetitions of the stimulus.

## Responses to Electrical Stimulation

Sensory information enters the hippocampus by two inputs. The subcortical input (via MS) introduces relatively “raw” information, while the cortical input (via the entorhinal cortex) conveys highly processed information. In order to separate the effects of theta activity on these inputs, the responses of theta interneurons to electric stimulation of respective afferents were analyzed. The analysis of these responses in control and during artificially changed theta oscillations can give some additional information about the role of cortical and subcortical inputs in signal processing[44,45].

The responses to low-frequency (1–10 Hz) stimulation of the MS and of the cortical inputs (perforant path for CA1 and mossy fibers for CA3 theta interneurons) were analyzed. Because the theta rhythm-dependent changes in responses to the stimulation of the perforant path and mossy fibers were similar, these data were pooled for the final analysis.

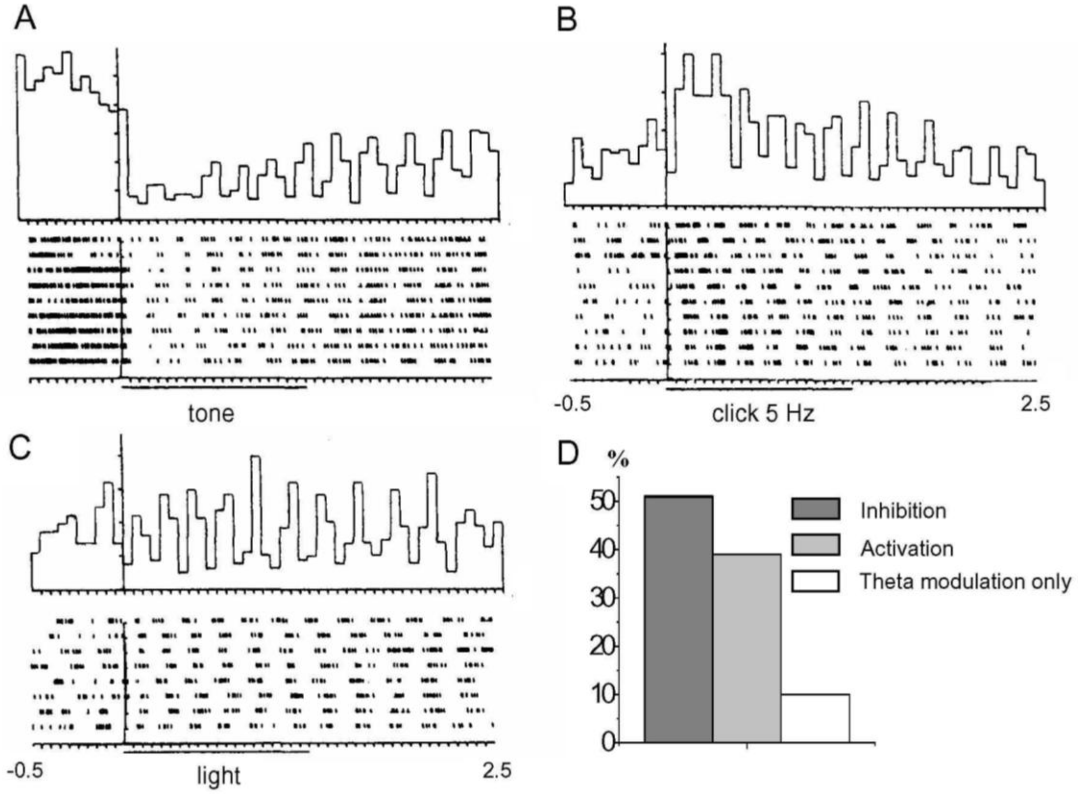

**FIGURE 2**. Sensory responses of theta hippocampal interneurons in the control (peristimulus time histograms and raster displays of a series of responses; X axis – time, sec; Y axis – the number of spikes/bin). (A) Inhibition evoked by a pure tone. (B) Excitation induced by a rhythmic click. (C) Increase of theta modulation without changes in the spiking rate evoked by light. One can see an augmentation of theta rhythmicity in spiking after a short inhibitory period (reset of theta activity) and phase-locking of theta bursts to stimuli. The vertical line on the plot indicates the moment of switching on the stimuli; the time of stimulus presentation is shown by lines below raster displays. (D) Distribution of different types of responses, % from all reactions.

## Responses to MS Stimulation

MS plays a key role in the generation of hippocampal theta rhythm[3,41]. In addition, it processes the information ascending from multiple structures of the brainstem and relays it to the hippocampus. Thus, analyses of hippocampal responses to MS stimulation and their changes after artificial alterations of theta oscillations have a special interest for understanding the role of this rhythmical activity in the brain information processes.

The majority (55%) of CA1-CA3 theta cells responded to the stimulation of MS by a pronounced initial suppression of activity; a smaller part (36.5%) of neurons reacted by prolonged excitation often accompanied by an enhanced theta modulation (Fig. 3A[a,b], left), or by burst of spikes (bursting on-response, 8.5%). When the theta activity was distinct ($\tau_\theta$ more than 0.3), the stimulation evoked a clear alternation of bursts and pauses, which phase-locked to stimulus (Fig. 3A[b], left). Repetitive MS stimulation resulted in the rhythmic driving of theta bursts or of initial inhibition; the stimulation with frequencies higher than 10 Hz led to ignoring the stimuli and return to baseline rhythmicity. Bursting on-responses could follow the stimuli with frequencies up to 20–25 Hz. Thus, stimulation of the MS evoked various reactions that may be due to the complexity of MS projections to the hippocampus (GABA, choline, and glutamatergic)[8,9,10,18,20,39]. Rhythmic driving of theta interneurons by MS stimulation suggests that MS probably provides phase-locking of theta cycles to the stimulus that was observed in sensory responses.

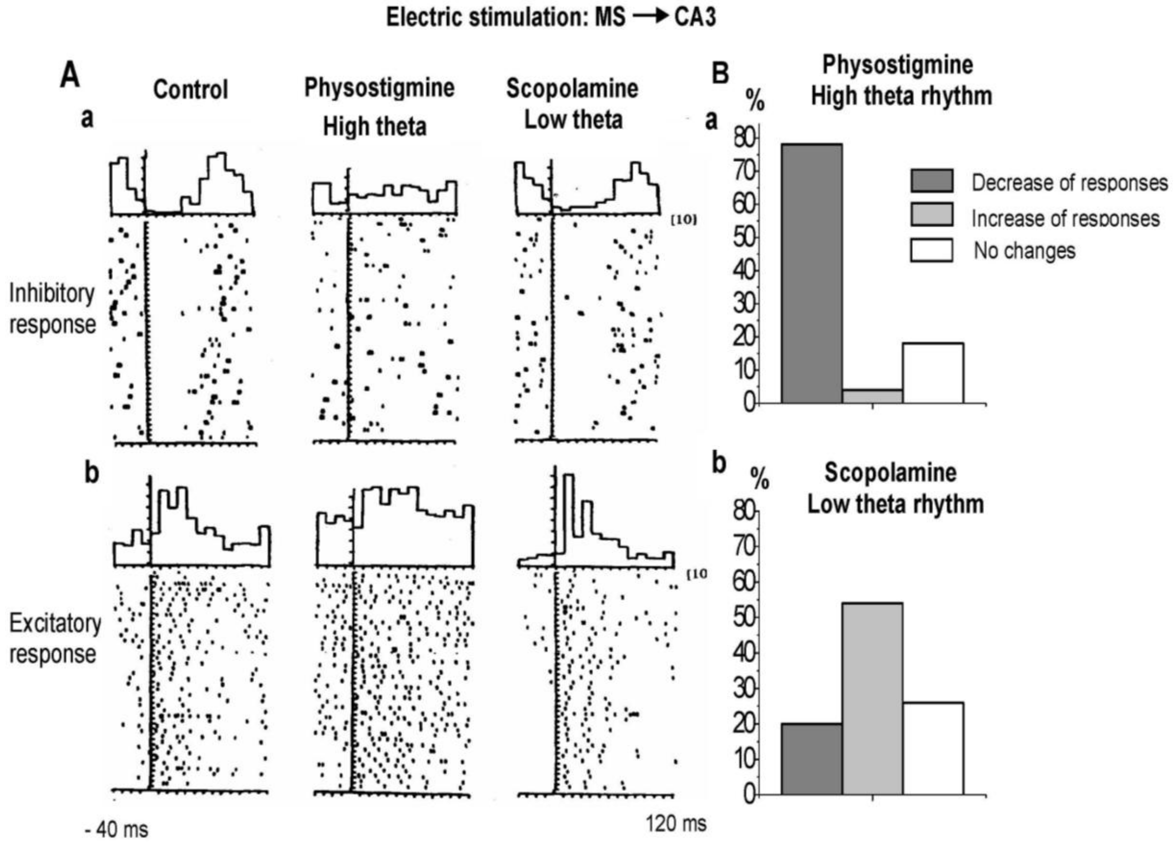

**FIGURE 3**. Responses of CA3 hippocampal interneurons to electrical stimulation of MS in control and after strong increase or decrease of theta rhythm. Peristimulus time histograms and raster displays of a series of responses to electrical 7-Hz stimulation of the MS are demonstrated (X axis – time, sec; Y axis – the number of spikes/bin; bin, 10 msec). (A) Responses of two neurons to MS stimulation: inhibitory (a) and excitatory response (b). One can see decrease/disappearance of responses after the injection of physostigmine and preservation/increase after the injection of scopolamine. (B) Changes of responses due to a strong increase (a) or blockade (b) of theta rhythm, % from all changes comparative to control.

## *Responses to Cortical Input (Perforant Path and Mossy Fibers) Stimulation*

Highly preprocessed complex information from the neocortex reaches the hippocampus through the perirhinal, entorhinal cortex, and presubiculum. These cortical areas gather information from the highest integrative levels – secondary and associative areas of posterior and anterior neocortex, which makes them the last stage of information processing in the cortical hierarchy[42]. The major part of cortical input (perforant path, PP) reaches CA3 after additional switching on the intrahippocampal relay structure – fascia dentata. The analysis of responses to PP and mossy fiber stimulation, and their changes due to enhanced or suppressed theta rhythm, is very important for understanding the role of theta activity in the brain information processes.

The majority of CA1-CA3 theta cells (82.5%) responded to cortical input stimulation by a group of spikes with a subsequent inhibition. Sometimes, especially in CA3, a frequency potentiation was observed when a series of stimuli with frequencies of 5–10 Hz was used (Fig. 4A[a,b], left). The responses of a minor group of theta interneurons (17.5%) consisted of an initial suppression of the activity; these responses may be produced by other inhibitory cells that receive direct input from the stimulated fibers and synapse on the recorded theta cell[9]. Thus, stimulation of the cortical inputs mostly caused a phasic excitatory response that may be followed by, or superimposed with inhibition, depending on the complexity of excited network.

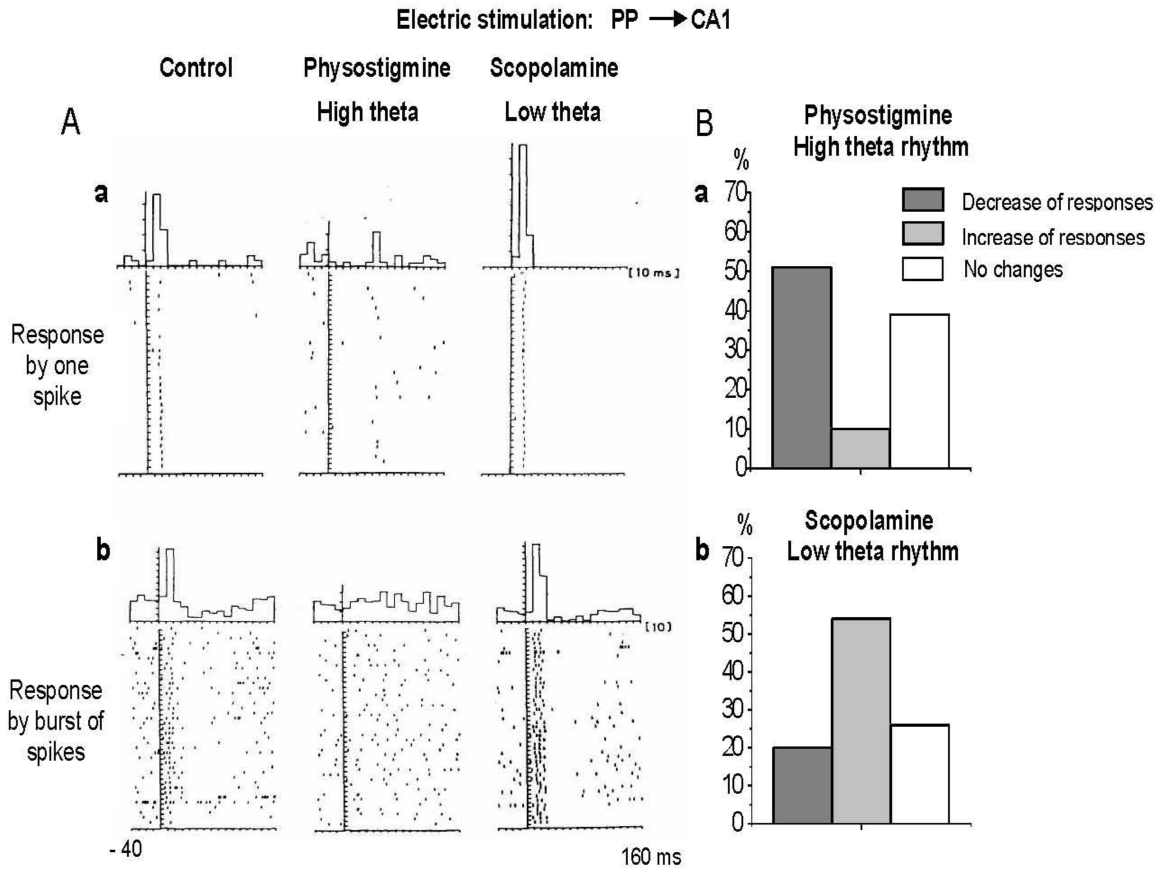

**FIGURE 4**. Responses of CA1 hippocampal interneurons to PP stimulation in the control and after strong increase or decrease of theta rhythm. Peristimulus time histograms and raster displays of a series of responses to electrical 5-Hz stimulation of the PP are showed (X axis – time, sec; Y axis – the number of spikes/bin; bin, 10 msec). (A) Single-spike (a) and burst (b) responses. (B) Changes of responses with a strong increase (a) or blockade (b) of theta rhythm, % from all changes comparative control.

## STRONG INCREASE OF THETA RHYTHM BLOCKS THE MAJORITY OF RESPONSES

One of the methods of theta increase was an intravenous injection of the acetylcholinesterase inhibitor physostigmine (0.1 mg/kg), a functional agonist of acetylcholine, which increased theta rhythm in the hippocampal EEG to 195 ± 82% of control values[43]. Another, more effective, way to theta elevation was an intraventricular injection of the alpha2 adrenoceptor agonist clonidine (5 µg dissolved in 5 µl), which evidently increased the adrenergic transmission in the brain[24]. Clonidine increased the hippocampal theta rhythm to 390 ± 89% of control. The third mode of theta increase was an injection of the local anesthetic lidocaine into the median raphe nucleus (0.25 µg in 2.5 µl) because, normally, theta rhythm is suppressed by the median raphe activity[22,47]. In this case, theta increased to 180 ± 89%. Changes of other parameters of the theta rhythm after injection of pharmacological drugs are presented in Table 1. These methods also increased the expression of theta bursting in a majority of recorded interneurons. In particular, regularity of bursts ($\tau_\theta$) increased from 0.29 ± 0.06 sec in the control to 0.45 ± 0.04 sec after physostigmine injection, to 0.61 ± 0.06 sec after clonidine, and to 0.42 ± 0.02 sec after delivery of lidocaine into the median raphe nucleus. Changes of other parameters of rhythmicity of theta cells after injection of pharmacological drugs are present in Table 1. In 8.3% of cells, the regularity of rhythmic burst values ($\tau_\theta$) increased to a degree ranging from 0.51 to 0.85 sec, which was never seen under control conditions (Figs. 1B,C; see also Table 1).

## Responses to Sensory Stimulation

Under all three conditions of increased theta rhythm, sensory responses had changed in 85% of theta cells; 40% of theta interneurons no longer responded to sensory stimuli. Inhibitory responses were especially affected: they were depressed (65%) or blocked (18%; Fig. 5B[a]), or even inverted (became excitatory; 10%) in 93% of cells with inhibitory responses in control. Excitatory responses were also affected; the dominating effect was a reduction of the magnitude of responses to sensory stimuli, which led to a decrease in the signal-to-noise ratio (Fig. 5B[b]). A detailed analysis revealed that the initial inhibition and phase-locking of theta bursts to sensory stimuli did not occur anymore (Fig. 5B[b]). However, in some theta cells, the responses were enhanced (22%), and the signal-to-noise ratio did not change or even increased. In these cases, the stimulus continued to reset the rhythmic activity via the initial inhibition, although the duration of this inhibition decreased compared to the control (75.0 ± 6.5 msec, $p < 0.01$). On the whole, the responses to sensory stimulation under increased theta conditions were completely or partly suppressed in 64% of theta interneurons (Fig. 5C). In 89% of still-responding cells, both inhibitory and excitatory sensory responses habituated after eight to 10 repetitions of the stimulus.

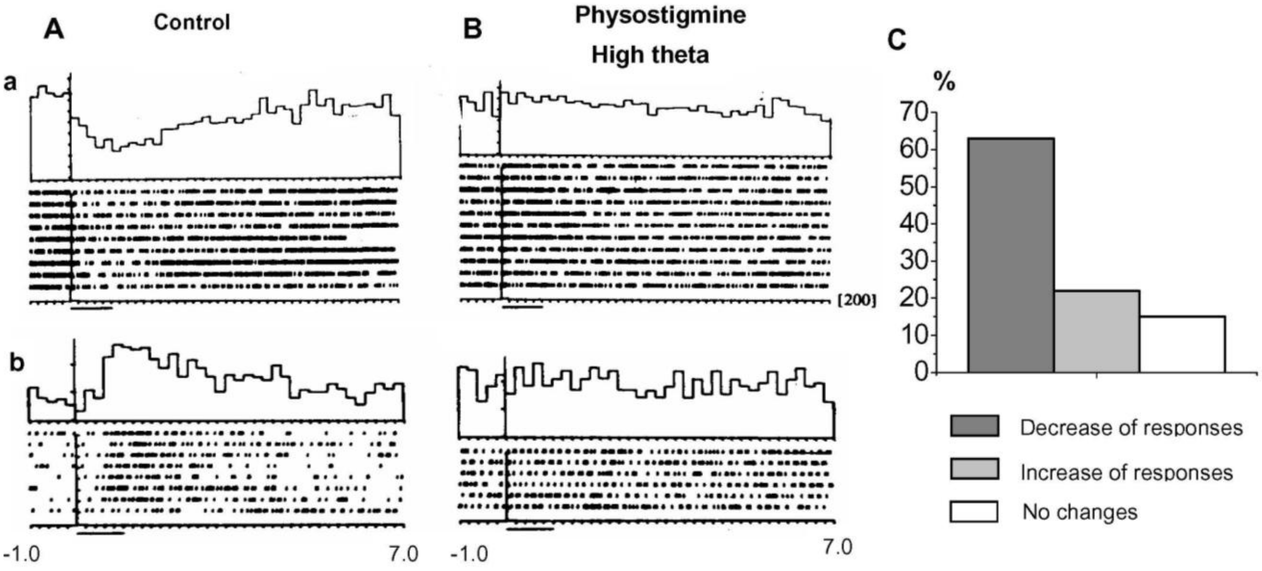

**FIGURE 5**. Changes of sensory responses of theta interneurons after a strong increase of theta modulation (physostigmine injection). (A) Inhibitory ([a] air puff) and excitatory ([b] rhythmic flash) sensory responses in control. (B) Disappearance of sensory responses after the injection of physostigmine. (C) Distribution of high theta rhythm effects on sensory responses, % from all changes comparative control. All marking as in Fig. 2.

## Responses to Electrical Stimulation

### *Effects of MS Stimulation*

Responses to MS stimulation were transformed in 82% of theta interneurons. The changes in theta cells exhibiting primary inhibitory responses were especially distinct and uniform; in 92%, the inhibitory responses disappeared or became weaker (Fig. 3A[a], middle). The changes in excitatory effects were less uniform, although a similar tendency was observed (Fig. 3A[b], middle). On the whole, the responses to MS stimulation under increased theta conditions were completely or partly suppressed in 78% of theta interneurons (Fig. 3B[a]). In many theta cells, MS stimulation could not reset theta bursting and they continued with their own rhythm without stimulus locking. Thus, the effect of phase-locking of theta cycles by MS stimulation was completely eliminated under enhanced theta conditions.

### *Effects of Cortical Input Stimulation*

In 52% of cells, responses to the stimulation of cortical inputs were abolished or significantly decreased. In some theta interneurons, the weakening of reaction manifested itself in a significant decrease in the phase-locking of theta bursts and the decrease or loss of frequency potentiation (Fig. 4A[a,b], middle). But in 38% of neurons, short excitatory reactions (*on*-responses) did not change, and in some cases (10% of cells), an increase of responses was observed. During electrical stimulation, the responses were preserved or enhanced only for the stimuli that, for some unknown reason, were able to reset the rhythmic activity (similar to the increased sensory responses, described above).

All these changes disappeared 30–40 min after the restoration of the control level of theta rhythm in the EEG and neuronal activity. Thus, irrespective of the experimental paradigm, a strong increase of theta led to a decrease in the responses of the majority of putative hippocampal theta interneurons to external stimulation.

## THETA RHYTHM BLOCKADE PRESERVES AND ENHANCES THE RESPONSES TO THE INPUT STIMULATION

Three different protocols were used to decrease the hippocampal theta rhythm. One of the methods was an intravenous injection of the muscarinic antagonist scopolamine (0.3–1.0 mg/kg), which decreased theta rhythm in the hippocampal EEG to 72 ± 12% of control[43]. The second method, an intraventricular injection of the alpha2 adrenoceptor antagonist idazoxan (100 μg/5 μl), decreased the theta rhythm in the hippocampus to 51 ± 12% (probably via the inhibition of adrenergic transmission[22,23]). The most effective mode of theta blockade was an injection of the local anesthetic lidocaine into the MS (0.25 μg/2.5 μl)[47]. In this case, theta disappeared completely. Changes of other parameters of the theta rhythm after injection of pharmacological drugs are presented in Table 1. A systemic delivery of scopolamine or idazoxan, or the injection of lidocaine into MS, led to a decrease in the expression of theta bursting in the majority of theta interneurons and the disappearance of rhythmic bursts in 11.5% of cells (Fig. 1C, bottom; see also Table 1).

### Responses to Sensory Stimulation

The changes in sensory responses with EEG theta rhythm suppression were in general much less prominent than those caused by the increase in theta rhythm. Sensory responses in 44% of neurons remained unchanged (Fig. 6C). The inhibitory responses were especially resistant to theta disappearance; sometimes they became more distinct or even greatly enhanced. The decreased theta rhythm had stronger effects on the excitatory responses; in 41% of theta cells, the magnitude of existing excitatory responses significantly increased or new excitatory responses appeared in previously unresponsive interneurons, partly due to an increase of the signal-to-noise ratio. The initial inhibitory period (reset) under theta blockade became longer (210 ± 8.5 vs. 108.6 ± 4.7 msec in control; $p < 0.01$). The most important phenomenon of decreased EEG theta was the disappearance of gradual habituation to repeated sensory stimuli; the majority of sensory responses (78%) were reproduced without decrement despite long periods of multiple repetitions (Fig. 6B). To compare, 82% of sensory responses habituated with repetitions under control conditions, and 89% during the enhanced theta rhythm.

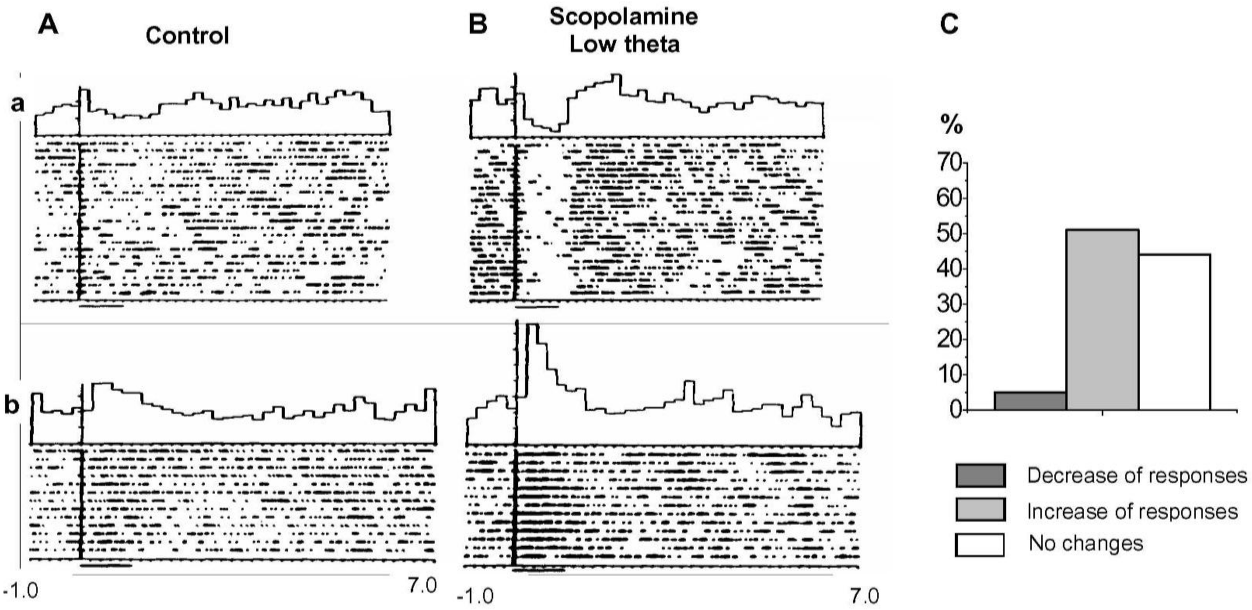

**FIGURE 6**. Changes of sensory responses after blockade of the theta rhythm (scopolamine injection). (A) Inhibitory ([a] pure tone) and excitatory ([b] light) sensory responses in control. (B) Increase in sensory responses after the injection of scopolamine. One can see that after injection of scopolamine, the responses do not habituate with repetition of stimuli, while in the control, habituation was distinct. (C) Diagram of changes of sensory responses after suppression of theta rhythm, % from all changes comparative control. All marking as in Fig. 2.

## Responses to Electrical Stimulation

### *Effects of MS Stimulation*

In half of the neurons, the responses to MS stimulation (54%) were retained or even increased under theta blockade conditions. The inhibitory responses were especially resistant: 68% of them did not change or became more distinct; often (29%), the responses to MS stimulation increased, sometimes due to an increase in the signal-to-noise ratio. Some excitatory responses (20%) were reversed; the stimulation now evoked a suppression of activity instead of excitation. The total proportion of hippocampal interneurons responding to MS stimulation did not differ from the control, but the responses often became more distinct (by an increase in the signal-to-noise ratio) (Fig. 3A[b], right). On the whole, the responses to MS stimulation under decreased theta conditions were increased in 54% of theta interneurons and decreased only in 20%; remaining cells (26%) did not change (Fig. 3B[b]).

### *Effects of Cortical Input Stimulation*

Responses to the stimulation of cortical inputs did not change in 47.5% of cells. Inhibitory responses and the inhibitory components of complex excitatory/inhibitory responses were retained or became more intense (for example, prolongation of the inhibitory phase) (Fig. 4A[a,b], right). Excitatory responses in 37.5% of theta interneurons significantly increased; their stability and/or intensity enhanced. In CA3 interneurons, frequency potentiation during the stimulation of mossy fibers was also often increased. Only in a few cases (7%) were the excitatory responses decreased or inverted into the inhibitory responses. On the whole, the responses to cortical input stimulation under decreased theta conditions were increased in 57.5% of theta interneurons and decreased only in 10%.

So, irrespective of the experimental approach used for EEG theta blockade, a decrease in the theta rhythm led to an increase in the responsiveness of the majority of putative hippocampal interneurons to all types of external stimulation.

## DISCUSSION

The hippocampus plays a key role in the recording of new information[28,41,42] and theta oscillations are believed to participate in this process[3,41]. How the rhythmic theta activity contributes to such an important brain function remains unknown. In our previous papers[22,44,45,46,47], we hypothesized that the functional role of hippocampal theta oscillations is the selection of signals for recording and storage. The examination of the activity of theta-bursting interneurons in the hippocampal stratum oriens appears consistent with this hypothesis.

According to experimental data, the hippocampal theta rhythm in control was changeable and response of theta interneurons to sensory stimulation habituated (disappeared) when the stimulus was repeated. New signals evoked a short inhibition of constant duration (reset) and subsequent phase-locking of hippocampal theta waves to the onset of signals associated with theta synchronization of field activity. This is consistent with the reset of theta EEG by electrical stimuli observed in another study[48]. We believe that only signals capable of evoking these changes are selected by the hippocampal system for recording and storage. When such a response to salient stimulus occurs, the following increased theta synchronization protects the hippocampal system from the interference with irrelevant signals, thus providing the correct processing of important information.

The absence of the theta deprives the hippocampus of this protection, which can lead to the distortion of signal coding[46]. As described above, the reset of theta bursting during the theta blockade was too long; the sensory responses became strong and invariable (did not habituate), which suggests that the system had lost the ability to select the salient signal for recording. In this state, the hippocampus lost the ability of “active ignoring” of insignificant stimuli[35].

During strong theta increases, in the majority of theta interneurons, sensory responses were decreased or disappeared. The resetting of the rhythmic theta activity and the phase-locking of theta cycles to stimuli were also impaired, although in a different way. During increased persistent theta, the sensory responses were essentially absent and the reset of theta bursting most often disappeared. The theta interneurons just continued bursting in their own rhythm without any relevance to stimuli. Therefore, the hippocampal system was probably closed and no information could be recorded.

As mentioned above, sensory information enters into the hippocampus by two inputs: subcortical and cortical. Acting as a comparator of the two information flows evoked by the same event[28,42], the hippocampus uses theta oscillations as a gating mechanism for the selection of the novel/important signals. The important instrument for this gating mechanism is evidently the reset of the going theta oscillations by the salient stimuli. What is the nature of the stimulus-induced initial inhibition in the hippocampus? It has been assumed that the short inhibition induced by sensory stimuli is septum dependent[44]. This supposition agrees with the results of other authors[11]. And what is more, it has been shown that this short inhibition was mediated by the GABAergic septal mechanism[1]. It is evident that this stimulus-induced pause in cell discharges represents a universal mechanism of the theta phase reset and theta synchronization of a great number of hippocampal cells. This appears to be necessary for the hippocampal function of selection of information for recording and storage.

Interestingly, a comparison of the spontaneous and evoked activities of hippocampal neurons during a strong increase in theta rhythm (regardless of the method of theta increase) revealed the similar types of dysfunction, while different types of dysfunction were observed when EEG theta was suppressed. These facts suggest that all changes in the hippocampal theta activity occur via a common septohippocampal pathway. As has been demonstrated earlier, a subset of stratum oriens hippocampal neurons is the main target of septal GABAergic cells[8,18]. These septal GABAergic neurons possess endogenous rhythmical properties and discharge in rhythmic bursts at theta frequency[21,36]. Besides GABAergic septal cells, cholinergic neurons also project from the MS to the hippocampal GABAergic interneurons[10]. A feedback output from the hippocampus to MS also originates principally from GABAergic neurons located in the stratum oriens of the CA1 and CA3 hippocampal regions[17,39]. These GABAergic septohippocampal interconnections could underlay the generation of hippocampal theta rhythm[40] and filter the hippocampal sensory responses, depending on the strength of theta rhythm.

The data suggest that nonpyramidal neurons in the stratum oriens participate in the selection of sensory signals by the hippocampal system. Among these may be the oriens-lacunosum moleculare (O-LM) interneurons because they are known to fire intermittently at the theta frequency[3,25]. O-LM interneurons are of great interest since they evidently provide a theta-frequency patterned output to distal dendritic segments of pyramidal cells[13] that receive specific sensory information (reviewed in Vinogradova[41]) essential for the activity of place cells[30].
